# Supplementary material for: Seasonality of respiratory, enteric, and urinary viruses revealed by wastewater genomic surveillance
Source: mSphere. 2024 May 7;9(5):e00105-24. doi: 10.1128/msphere.00105-24 (PMC11237574; doi:10.1128/msphere.00105-24)
Supplement: Figure S1 — Plots. [file msphere.00105-24-s0001.pdf]

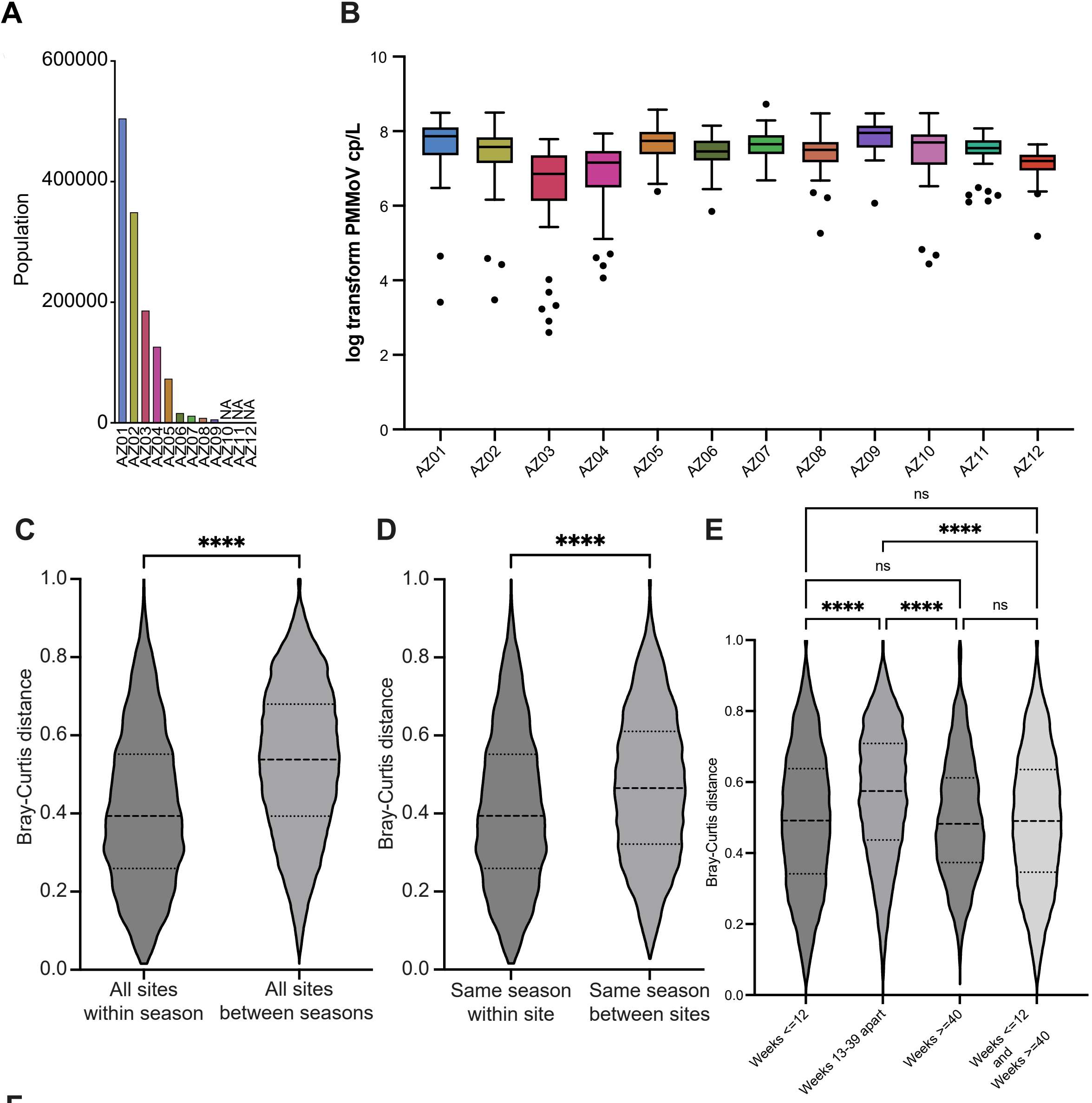

F

groups	control	case	coefficient	p-value	padjust	interpretation
1v4	Fall	Spring	5.75	0.00	0.00	There is more fall then spring in group4 in comparison to group1
1v4	Fall	Summer	2.30	0.01	0.01	There is more fall then summer in group4 in comparison to group1
1v4	Fall	Winter2021	6.80	0.00	0.00	There is more fall then winter2021 in group4 in comparison to group1
1v4	Fall	Winter2022	2.40	0.01	0.01	There is more fall then winter2022 in group4 in comparison to group1
1v4	summer	Winter2021	4.51	0.00	0.01	There is more summer than winter2021 in group4 in comparison to group1
1v4	Spring	Summer	-3.46	0.00	0.00	Three is less spring then summer in group4 in comparison to group1
3v1	Fall	Spring	-5.10	0.00	0.00	There is less fall then spring in group2 in comparison to group 3
3v1	Fall	Winter2021	-5.79	0.00	0.00	There is less fall then winter2021 in group2 in comparison to group3
3v1	summer	Winter2021	-4.82	0.00	0.00	There is less summer than winter 2021 in group2 in comparison to group3
3v1	Spring	Summer	4.13	0.00	0.00	There is more spring then summer in group2 in comparison to group3
3v1	Spring	Winter2022	4.59	0.00	0.01	There is more spring then winter2022 in grup in comparison to group3
3v1	Winter2021	Winter2022	5.28	0.00	0.01	There is more winter2021 then winter22 in group2 then group 3
3v4	Fall	Summer	2.56	0.00	0.01	There is more fall then summer in group1 incomparison to group4
3v4	Spring	Summer	2.00	0.01	0.01	There is more spring then summer in group1 in comparison to group4
2v1	Fall	Spring	-4.73	0.00	0.01	There is less fall then spring in group2 in comparison to group 4
2v1	summer	Winter2021	-6.06	0.00	0.00	There is less summer then winter2021 in group2 then group4
2v1	Spring	Summer	5.23	0.00	0.00	There is more spring then summer in group2 in comparison to group4
2v1	Winter2021	fall	5.55	0.00	0.01	There is more winter2021 then fall in group2 in comparison to group4
2v1	Winter2022	fall	2.40	0.01	0.01	There is more winter2022 then fall in group2 in comparison to group4
3v4	summer	Winter2022	-2.66	0.01	0.01	There is less summer then winter22 in group3 in comparison to group 4

Supplementary Figure S1. (A) Population estimates for catchments represented by each sampling site. (B) Box-plots of PMMoV viral load in wastewater (genome copies per liter wastewater) for each site. (C) Violin plot of bray-curtis distances of all sites by same season vs. different seasons. Statistical significance assessed by Mann-Whitney. (D) Violin plot of bray-curtis distances of same sites vs. different sites, controlled by same season. Statistical significance assessed by Mann-Whitney. (E) Bray-curits distances plotted for samples 1-12 weeks apart, 13-39 weeks apart, more than 40 weeks apart and combining first and third violin plot values. Statistical significance assessed by Kruskal Wallis with corrections for multiple comparisons. (F) Table for significant comparisons done using mclogit results for k-means clusters and metrological seasons.
